# Discovery of novel choline acetyltransferase inhibitors using structure-based virtual screening

**DOI:** 10.1038/s41598-017-16033-w

**Published:** 2017-11-24

**Authors:** Rajnish Kumar, Amit Kumar, Bengt Långström, Taher Darreh-Shori

**Affiliations:** 1 Center for Alzheimer Research, Karolinska Institutet, Department of Neurobiology, Care Sciences and Society, Division of Translational Alzheimer Neurobiology., NOVUM, 4th Floor, 141 86 Stockholm, Sweden; 2 Department of Chemistry, Uppsala University, Uppsala, Sweden

## Abstract

Alzheimer disease and related dementias are major challenges, demanding urgent needs for earliest possible diagnosis to optimize the success rate in finding effective therapeutic interventions. Mounting solid scientific premises point at the core acetylcholine-biosynthesizing cholinergic enzyme, ChAT as a legitimate *in vivo* target for developing positron emission tomography biomarker for early diagnosis and/or monitoring therapeutic responses in the neurodegenerative dementias. Up-to-date, no PET tracer ligands for ChAT are available. Here we report for the first time a novel hierarchical virtual screening approach on a commercial library of ~300,000 compounds, followed by *in vitro* screening of the hits by a new High-Throughput ChAT assay. We report detailed pharmacodynamic data for three identified selective novel ChAT ligands with IC_50_ and *K*_*i*_ values ranging from ~7 to 26 µM. In addition, several novel selective inhibitors of the acetylcholine-degrading enzymes, AChE and BuChE were identified, with one of the compounds showing an IC_50_-value of ~6 µM for AChE. In conclusion, this report provides an excellent starting platform for designing and optimizing potent and selective ChAT ligands, with high potential as PET-imaging probe for early diagnosis of AD, and related dementias, such as Down’s syndrome and Lewy body disorders.

## Introduction

At present, dementia is the major cause of death affecting approximately 47.5 million people worldwide and this figure is projected to be double by 2030^1^. Alzheimer’s disease (AD) type dementia alone prevalent in nearly 60–70% cases and designated as a “major killer”^2^. Other forms include dementia with Lewy bodies (DLB), frontotemporal dementia and vascular dementia. Beside almost a century of research in this field, there is no treatment available to cure the disease and only symptomatic treatments are available mainly indicating the use of acetylcholinesterase inhibitors to increase the availability of acetylcholine (ACh) in the diseased brain.

*In vitro* positron emission tomography (PET) imaging is gaining immense clinical impact and is an invaluable scientific tool for understanding the early pathological events in neurodegenerative disorders. It is also essential for effective monitoring of novel therapies and early diagnosis of neurodegeneration in AD^3^. In last few decades, increased number of labeled amyloid beta (Aβ) imaging agents based on conjugated Aβ specific dyes such as Congo red, thioflavin-T and PIB were developed and successfully tested for clinical diagnosis of AD^4^. However, as many as 30% of healthy elderly subjects with no clinical signs of dementia show PIB-retention in the brain. Whilst, some patients with no PIB-retention in the brain show severe cognitive deficits^5^. Aβ deposition is also a feature of DLB brain. Thereby, new more suitable *in vivo* PET biomarkers for a better disease prognostic and therapeutic evaluation are desirable.

Choline acetyltransferase (ChAT) (EC: 2.3.1.6; Choline O-acetyltransferase) is an important enzyme catalyzing the transfer of acetyl group from Acetyl-CoA to choline for synthesis of acetylcholine (ACh), which is a major neurotransmitter in the brain.

The neurons expressing ChAT are called cholinergic neurons and their communication with target tissues such as muscles depends on the functional ChAT. It has been observed that there is a decreased ChAT expression and activity in AD^6^. Therefore, ChAT has been proposed as a legitimate biomarker for early detection of AD and other neurodegenerative dementia disorders. Thus a PET tracer that can specifically bind to ChAT and help to monitor the health of cholinergic neurons will provide an important tool for early prognosis of AD.

The availability of a potent and specific ChAT radiotracer can be of significant interest in elucidating the functional role of this enzyme in the brain as well as in the peripheral system specifically related to cholinergic signaling in anti-inflammatory pathways and cancer biology. Unfortunately, few inhibitors of ChAT have been synthesized and reported so far, and mainly includes naphthyl-vinylpyridine derivatives, stilbazole derivatives, alkylaminoethyl esters and α-NETA^7^. The most studied class is napthylvinylpyridines, and their structure-activity relationships (SAR) studies identified three basic requirements for a potent ChAT inhibitor, which includes: 1) a cationic terminal on the amine end of molecule; 2) an aryl moiety on the acyl or keto end of the molecule; and 3) a partial positive charge on the carbon adjacent to the aryl moiety^8^. To be an effective ChAT inhibitor, the compound should also be highly potent, permeable to blood-brain barrier (BBB), and selective to ChAT as compared to other enzymes such as acetylcholinesterase (AChE) and butyrylcholinesterase (BuChE). The major limitation of the potent compounds till now is quaternary ammonium characteristics, which makes them impermeable to BBB and thus poses limited applicability.

Efforts were made to overcome this limitation by further replacing the amine-pyridine moiety with a heterocyclic amine like oxazine, and a potent inhibitor was identified^9^. So far, more than a hundred compounds have been reported as ChAT inhibitors, but none of them was able to achieve the desired *in vivo* efficacy and showed promises as a PET tracer.

In past few years, virtual screening has been evolved as a crucial part of pre-clinical drug discovery and has shown very encouraging result in the identification of early hits and lead compounds. The crystal structure of human ChAT have been reported and the catalytic site comprising of histidine amino acid (HIS_324_), is located in the center of the catalytic tunnel where choline binds at one end and Coenzyme A (CoA) binds to the another end^6,10^. The availability of crystal structure of ChAT gives an opportunity to use the virtual screening methods to identify new ligands of ChAT. In this paper, we report a novel virtual screening method targeted at small molecule chemical library for the discovery of potential ChAT inhibitors using structure-based molecular docking approach. Initially, the library was filtered for optimal physicochemical and pharmacokinetic properties of CNS acting drug such as Blood Brain Barrier (BBB) permeation, followed by Surflex-Screen docking. The top compounds from this screen were subjected to exhaustive Surflex-Dock GeomX protocol and the molecules were ranked according to their total score. After visual inspection of the top scoring compounds, 35 compounds were purchased from the vendor and tested *in vitro* for their inhibition potency using our newly developed, simple, robust and inexpensive non-radioactive in-house enzymatic assay method for the recombinant human ChAT (rChAT) enzyme. Enzyme kinetics and *in vitro* cellular toxicity studies were also performed in order to assess the druggability of identified compounds.

## Results

### Structure based virtual screening of Asinex Gold/Platinum small molecule library

**Figure 1:**
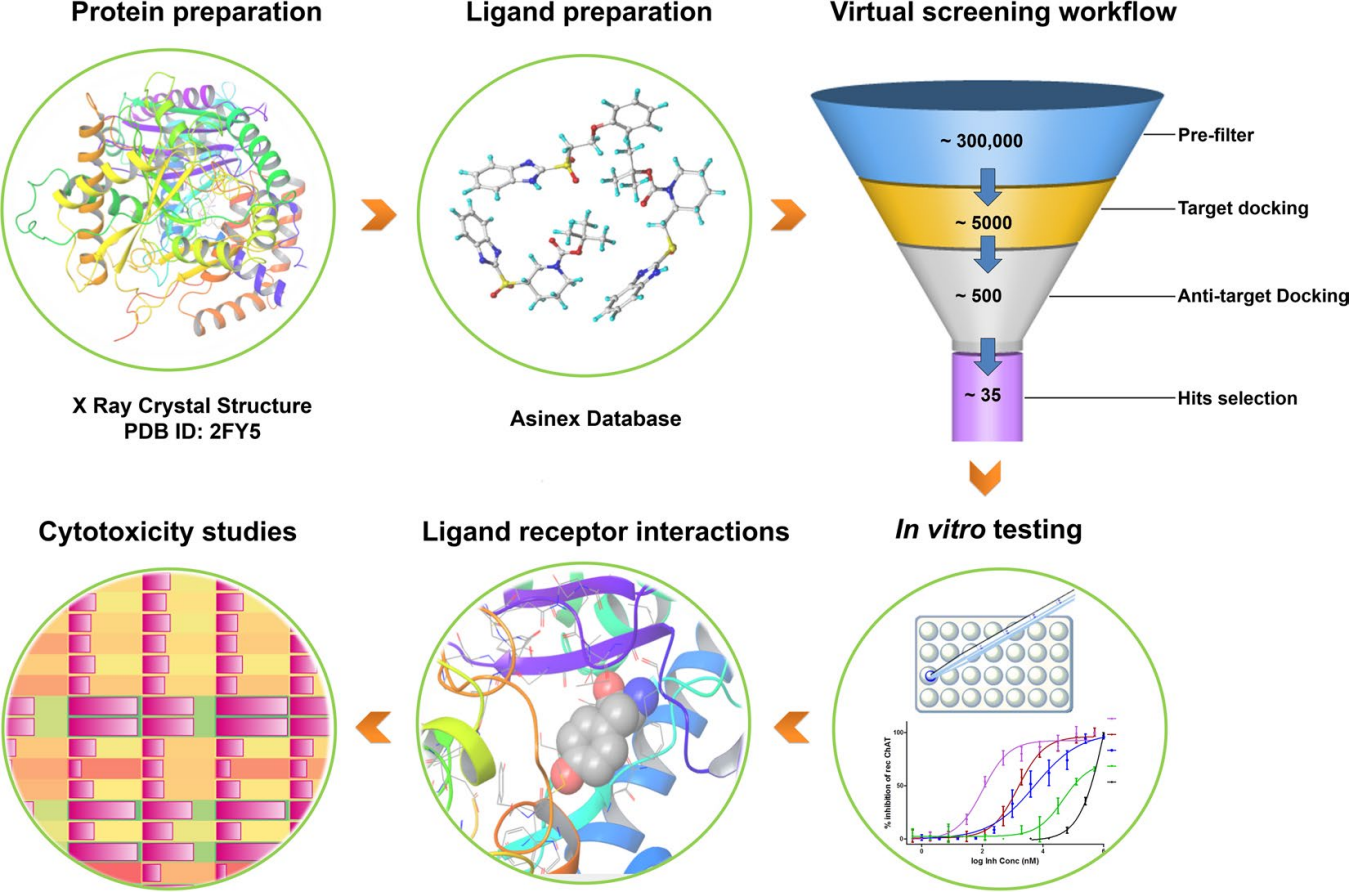
Overall workflow of the structure based virtual screening based identification of novel ChAT inhibitors. The protein structure was prepared followed by preparation of Asinex Gold/Platinum library comprising 296537 compounds. After an initial filtering of compounds for optimal physicochemical properties, the library was screened using Surflex-Dock docking algorithm. The compounds with a cutoff score of 7 were further subjected to flexible docking with Surflex-GeomX approach. The top scoring compounds were inspected visually for their molecular interaction and were procured and tested *in vitro* using our in house developed non-radioactive assay method. Additionally, *in vitro* cellular cytotoxicity studies were also performed.

**Figure 2:**
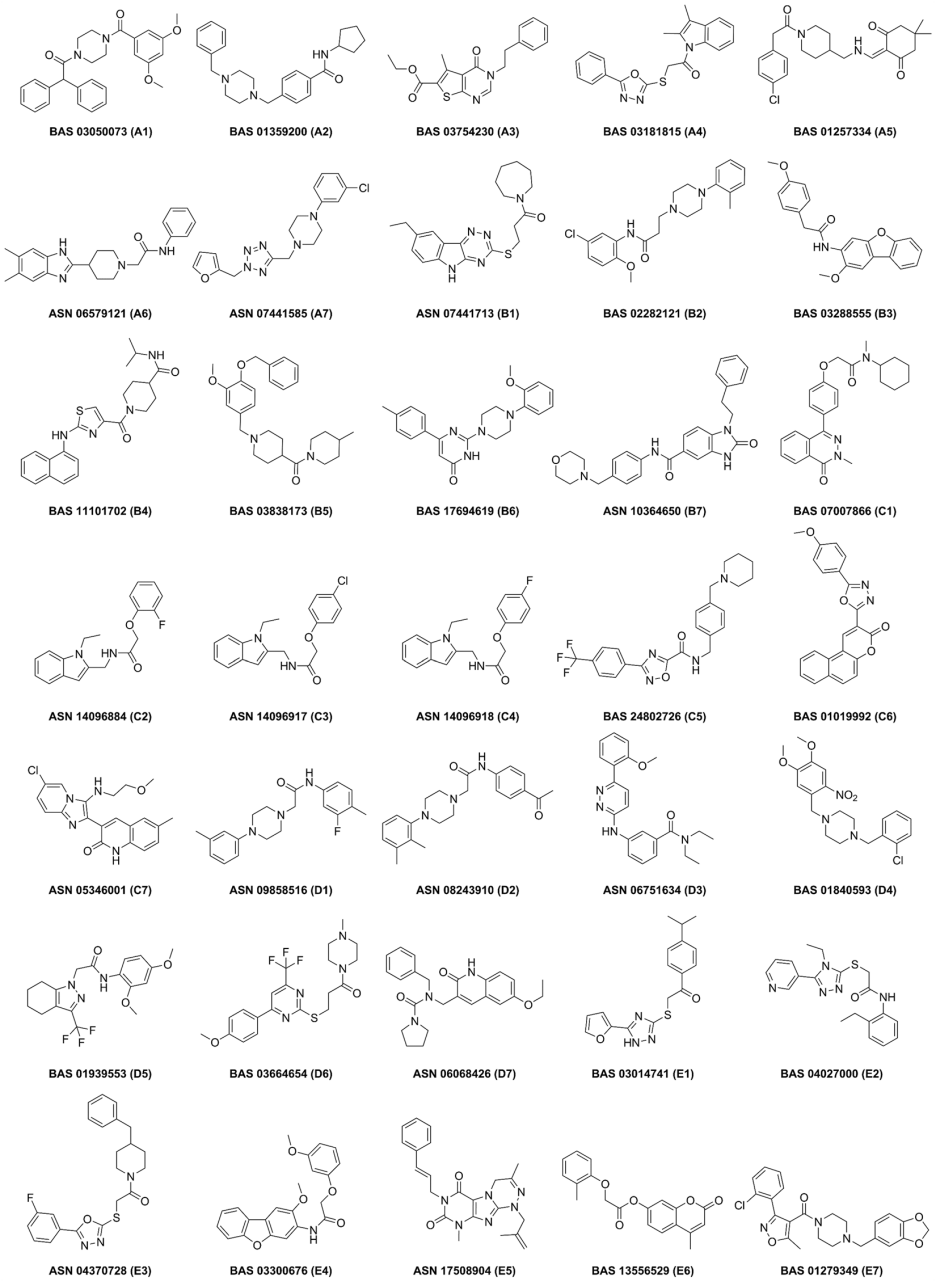
2D structure of 35 virtual screening based selected compounds from Asinex library. Out of the top scoring five hundred compounds, these 35 compounds were selected based on manual inspection of the interaction with the active site of ChAT. The Asinex compound ID number are given together with the compound code assigned by us in the parenthesis.

The ultimate aim of present study was to identify novel and specific ChAT ligands with optimal blood-brain barrier (BBB) permeability, which can be radiolabeled and used in order to map the enzyme concentration level in the brain. Therefore, our first step in the hierarchical virtual screening approach (Fig. 1) was to filter the dataset for compounds with potential BBB permeability. After an initial filtration of the Asinex library compounds (~300,000) on the basis of optimum physicochemical properties, based on modified Lipinski’s rule of five^11^ to ensure BBB permeability and excluding the undesirable and possible pan assay interference compounds (PAINS)^12^, a final dataset of 99823 compounds was obtained which was used for further Surflex-Dock GeomX based virtual screening. Following a combined approach using binding site of choline, acetyl coenzyme-A, and the common site that is represented by the catalytic His324 amino acid residue. Additionally, the top scoring compounds for ChAT were docked against anti-targets, AChE and BuChE and finally, 35 compounds were selected (Fig. 2) based on their low score for AChE and BuChE and high score for ChAT. The top scoring compounds along with their Surflex-Dock score representing −logK_d_, for ChAT, AChE and BuChE is given in Table 1.

**Table 1:** Surflex-Dock score of virtual screening based identified compounds against ChAT, AChE and BuChE along with cLogP and cLogD.

Compound ID	cLogP	cLogD^#^	ChAT			AChE			BuChE		
			Total Score^a^	Crash^b^	Polar^c^	Total Score^a^	Crash^b^	Polar^c^	Total Score^a^	Crash^b^	Polar^c^
A1	4.0	3.5	9.1	−2.9	3.7	−0.8	−14.5	0.0	6.5	−1.7	0.0
A2	4.4	3.2	9.3	−2.4	2.0	2.1	−6.8	0.0	5.3	−1.6	0.0
A3	3.5	3.9	9.8	−1.1	2.5	4.4	−4.8	1.0	6.2	−1.7	1.2
A4	4.2	3.8	9.5	−1.8	1.2	6.7	−2.9	0.3	6.1	−0.8	0.4
A5	3.5	3.2	9.1	−3.1	2.9	−0.9	−11.7	0.6	6.4	−2.6	1.2
A6	3.9	4.2	9.2	−2.0	2.4	0.8	−6.1	1.4	5.7	−1.4	0.0
A7	3.2	3.4	9.2	−1.8	2.5	3.6	−4.0	0.1	6.2	−0.7	0.3
B1	4.4	3.8	9.2	−2.5	3.2	2.3	−1.4	0.0	6.1	−3.6	1.6
B2	4.4	3.4	10.1	−2.9	2.9	2.4	−9.6	0.1	5.8	−1.2	0.0
B3	4.1	3.9	9.6	−2.1	1.3	1.4	−6.0	0.4	6.2	−0.5	0.0
B4	2.7	3.7	9.5	−4.0	2.2	−0.8	−12.5	0.6	6.1	−4.5	0.0
B5	3.8	3.6	9.1	−3.4	1.1	−12.9	−24.2	0.0	5.6	−1.7	0.0
B6	3.4	3.1	9.7	−2.1	1.6	0.2	−5.7	0.0	5.5	−1.5	0.0
B7	4.2	3.8	9.4	−2.3	1.3	−5.7	−17.5	0.1	6.0	−1.3	0.9
C1	4.0	3.7	9.0	−0.9	1.5	2.9	−9.0	0.4	6.7	−1.5	0.0
C2	3.8	3.2	9.2	−2.2	2.1	5.5	−3.7	0.5	6.9	−0.8	1.2
C3	4.5	3.6	9.5	−1.9	2.7	5.4	−3.7	1.0	6.6	−1.8	1.2
C4	4.0	3.2	9.4	−2.1	2.0	4.6	−6.0	0.9	6.4	−1.5	2.1
C5	5.4	3.3	9.2	−1.7	1.8	1.7	−4.8	0.4	5.5	−1.2	0.0
C6	3.6	3.7	9.9	−1.2	2.1	1.3	−4.3	2.0	5.3	−1.9	0.7
C7	4.2	3.7	9.5	−2.0	0.8	5.2	−4.8	1.9	6.7	−0.8	2.1
D1	4.6	4.0	9.3	−2.2	3.2	2.7	−7.5	0.5	6.6	−2.2	0.0
D2	4.1	3.4	9.3	−2.5	1.8	−1.8	−14.9	1.9	6.9	−0.9	0.8
D3	3.5	3.7	9.1	−1.5	1.7	1.1	−10.4	0.1	6.0	−5.0	0.0
D4	4.5	−1.7	10.1	−0.9	4.9	0.1	−10.3	1.7	5.9	−3.2	2.3
D5	2.9	3.5	10.0	−1.6	1.6	6.9	−3.9	0.7	6.8	−0.7	0.0
D6	3.8	3.4	9.3	−3.3	3.5	2.5	-8.8	0.6	6.7	−3.0	1.5
D7	3.4	3.9	9.1	−1.7	2.2	2.9	−10.2	1.3	6.5	−1.9	1.3
E1	4.7	3.9	9.1	−1.0	3.6	5.6	−3.7	1.2	6.7	−0.9	0.2
E2	1.7	3.2	9.5	−2.8	2.3	3.9	−3.3	1.2	6.1	−1.5	0.9
E3	3.5	4.0	9.0	−1.9	1.1	4.0	−6.4	0.5	5.5	−1.8	1.1
E4	4.2	3.6	10.1	−2.1	2.0	3.1	−8.4	0.8	6.1	−1.5	1.7
E5	4.1	3.3	9.1	−3.3	0.4	2.7	−4.7	0.0	5.8	−5.9	0.0
E6	3.6	3.7	9.2	−1.5	2.2	4.4	−4.3	0.0	7.0	−1.3	0.8
E7	4.4	3.7	9.2	−2.7	3.1	0.3	−11.1	0.8	6.0	−2.8	0.9

^a^Total Score is the total Surflex-Dock score expressed as -log(Kd)^36^. ^b^Crash is the degree of inappropriate penetration by the ligand into the protein and of interpenetration (self-clash) between ligand atoms that are separated by rotatable bonds. Crash scores close to 0 are favorable. ^c^Polar is the contribution of the polar interactions to the total score. ^#^Marvin was used for calculating pKa/Log D @ pH 7.4 (ClogD) values for the compounds, Marvin 15.4.13.0, 2015, ChemAxon (http://www.chemaxon.com). ChAT =  choline acetyltransferase; AChE = acetylcholinesterase; BuChE = butyrylcholinesterase.

### *In vitro* screening of the 35 hit compounds for ChAT inhibitory activities

**Figure 3:**
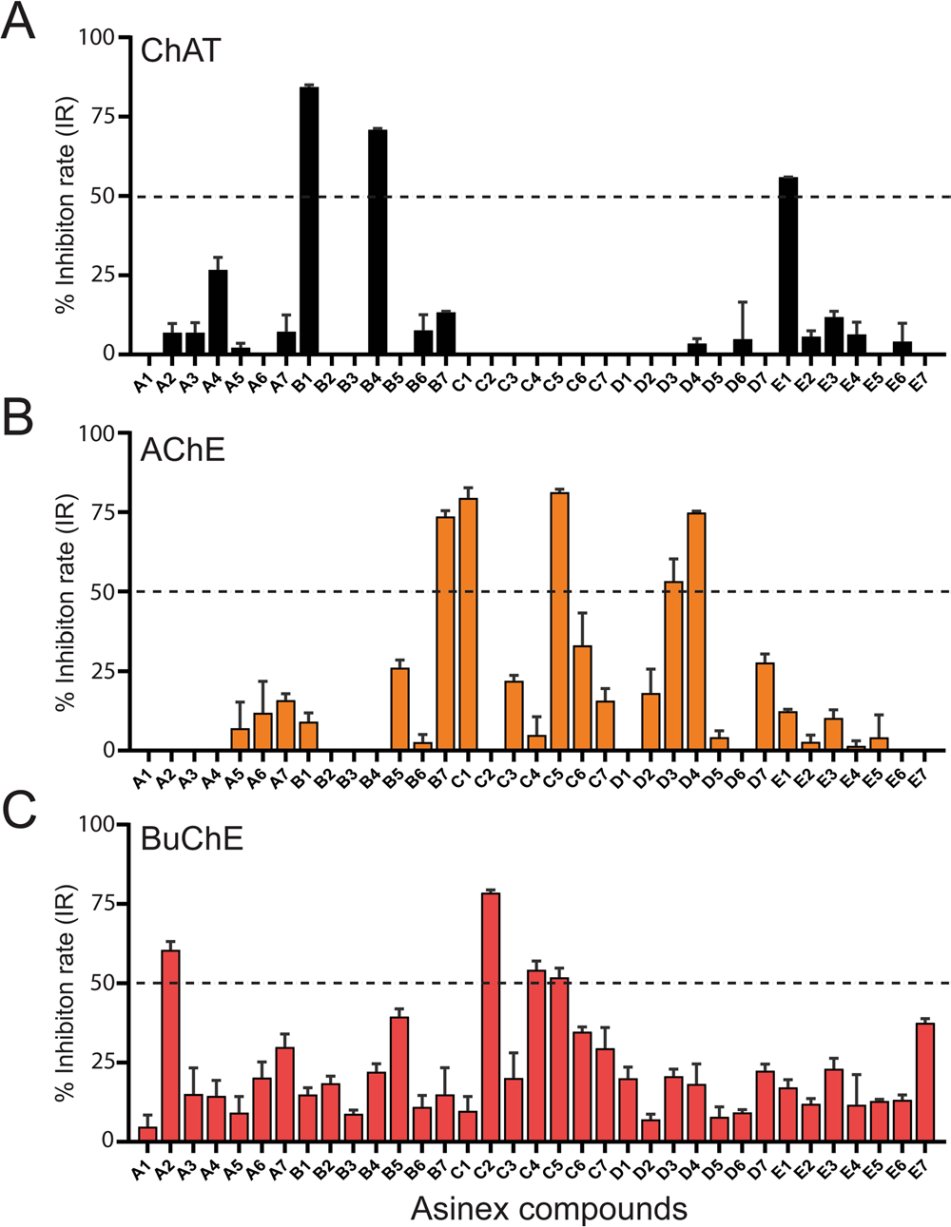
Screening of top 35 Asinex hit compounds for ChAT, AChE and BuChE activity inhibition. (**A**) Human rChAT was pre-incubated with 100 μM of different Asinex compounds for 10–30 minutes at room temperature. The activity was measured using our in-house developed  fluorometric assay. (**B**,**C**) rAChE & BuChE were pre-incubated with 200 μM of different Asinex compounds for 10–30 minutes at room temperature. The activity was measured using modified version of Ellman’s colorimetric assay. The effect of these compounds on rChAT, AChE and BuChE was compared to the activity of a control sample that was pre-incubated with the buffer alone. The data is presented as mean ± SD of three individual experiments performed in triplicate. The dashed lines are representing 50% inhibition rate (IR) value; the compound with over 50% IR were selected for further kinetic analysis. rChAT = recombinant choline acetyltransferase; rAChE = recombinant acetylcholinesterase; BuChE = butyrylcholinesterase.

At first, 35 identified hits from the virtual screening protocol, were procured and screened *in vitro* for their inhibition of human rChAT enzyme activity using our new in-house high throughput fluorometric assay, at a single concentration of 100 µM. As a positive control for the compounds, the only commercially available ChAT inhibitor α-NETA^7^ was included which resulted in 93% inhibition of the enzyme. This initial screening assay identified a total of three compounds: **B1**, **B4**, and **E1** for human rChAT with more than 50% inhibition of enzyme activity at the tested concentrations (Fig. 3; Table 2). All these compounds were selected and further assayed for their dose-dependent inhibitory activity and mode of inhibition determination.

**Table 2:** *In vitro* inhibitory activity evaluation of identified top 35 Asinex hit compounds on human rChAT, rAChE and plasma BuChE.

Compounds ID	ChAT			AChE			BuChE		
	% IR^a^	IC_50_^c^ (µM)	Ki^d^ (µM)	% IR^b^	IC_50_^c^ (µM)	Ki^d^ (µM)	% IR^b^	IC_50_^c^ (µM)	Ki^d^ (µM)
A1	0.0	—	—	0.0	—	—	4.8	—	—
A2	6.9	—	—	0.0	—	—	**60**.**5**	**526**.**9**	**84**.**9**
A3	6.9	—	—	0.0	—	—	15.1	—	—
A4	26.7	—	—	0.0	—	—	14.4	—	—
A5	2.1	—	—	7.0	—	—	9.2	—	—
A6	0.0	—	—	11.9	—	—	20.2	—	—
A7	7.2	—	—	15.8	—	—	29.9	—	—
B1	**84**.**3**	**7**.**0**	**9**.**4**	9.1	—	—	14.8	—	—
B2	0.0	—	—	0.0	—	—	18.5	—	—
B3	0.0	—	—	0.0	—	—	16.6	—	—
B4	**70**.**8**	**16**.**5**	**11**.**9**	0.0	—	—	22.1	—	—
B5	0.0	—	—	26.1	—	—	39.5	—	—
B6	7.6	—	—	2.6	—	—	11.0	—	—
B7	13.3	—	—	**73**.**6**	**40**.**1**	**65**.**4**	14.9	—	—
C1	0.0	—	—	**79**.**5**	**71**.**9**	**42**.**3**	9.8	—	—
C2	0.0	—	—	0.0	—	—	**78**.**5**	**364.5**	**59**.**6**
C3	0.0	—	—	22.0	—	—	20.1	—	—
C4	0.0	—	—	4.9	—	—	**54**.**2**	**527**.**5**	**59**.**4**
C5	0.0	—	—	**81**.**3**	**6**.**3**	**23**.**7**	51.8	—	—
C6	nd^e^	—	—	33.1	—	—	34.7	—	—
C7	0.0	—	—	15.7	—	—	29.4	—	—
D1	0.0	—	—	0.0	—	—	20.0	—	—
D2	0.0	—	—	18.1	—	—	7.0	—	—
D3	0.0	—	—	**53**.**2**	**250**.**8**	**177**.**1**	20.7	—	—
D4	3.5	—	—	**74**.**8**	**145**.**9**	**141**.**1**	18.2	—	—
D5	0.0	—	—	4.2	—	—	7.8	—	—
D6	4.8	—	—	0.0	—	—	9.3	—	—
D7	0.0	—	—	27.7	—	—	22.4	—	—
E1	**55**.**2**	**25**.**4**	**15**.**2**	12.4	—	—	17.2	—	—
E2	4.1	—	—	2.7	—	—	12.0	—	—
E3	10.3	—	—	10.2	—	—	23.0	—	—
E4	4.8	—	—	1.5	—	—	11.7	—	—
E5	0.0	—	—	4.1	—	—	12.8	—	—
E6	2.5	—	—	0.0	—	—	13.2	—	—
E7	0.0	—	—	0.5	—	—	37.5	—	—
α-NETA	93.7	0.1*	—	—	—	—	—	—	—

^a^% Inhibition rate (IR) at a concentration of 100 µM for rChAT. ^b^% IR at a concentration of 200 µM for rAChE and BuChE. % IR values are shows as mean of three individual experiments performed in triplicate. Results expressed as percentage inhibition compared to control. ^c^The half maximal inhibitory concentration (IC_50_) of the compounds to produce 50% inactivation of the enzymes; the values are shown as mean of two experiments. ^ d ^The inhibitory constant (Ki) values indicate the potency of identified inhibitors; the values are shown as mean of two experiments. ^e^Compound C6 interfered with ChAT fluorometric assay because of its colored nature. *As previously reported by our group^ 26^. α-NETA = 2-(alpha-naphthoyl)ethyltrimethylammonium iodide; rChAT = recombinant choline acetyltransferase; rAChE = recombinant acetylcholinesterase.

### *In vitro* estimation of inhibition constant and mode of inhibition for human rChAT hits

**Figure 4:**
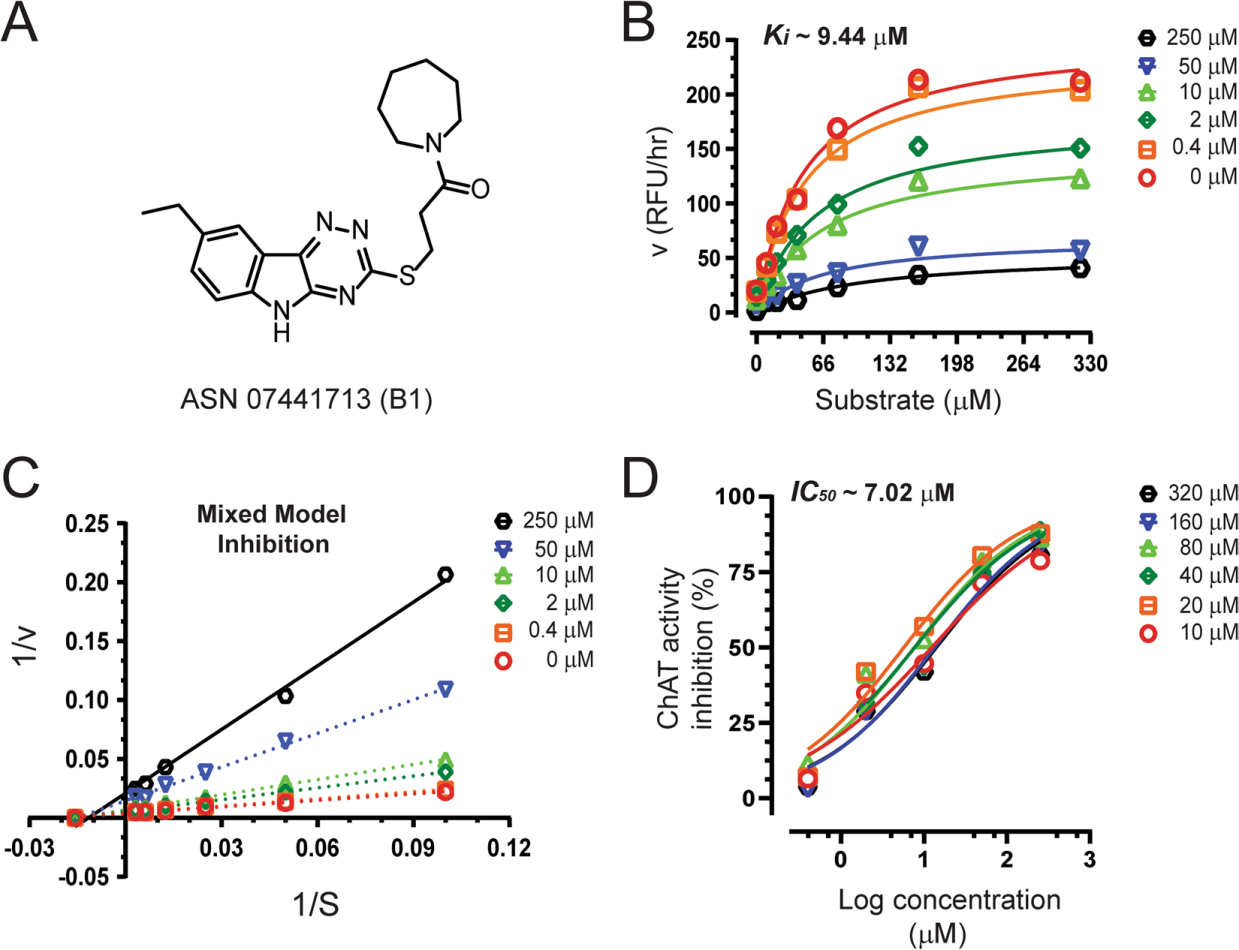
Inhibitory studies of compound **B1** against human rChAT. The specified concentrations of compound **B1** were pre-incubated with human rChAT and different substrate concentration at room temperature for 10–30 min. Afterwards; the activity was measured using our in-house non-radioactive fluorescence assay as described in the methods section. The effect of these compounds on rChAT was compared to the activity of a control sample that was pre-incubated with the buffer alone. (**A**) Structure of Asinex compound **B1**. (**B**) Showing the Substrate-velocity inhibition curves of human rChAT activity at different concentrations of **B1**. (**C**) The Lineweaver-Burk plot showing the mode of inhibition of compound **B1**. The plot was obtained from the substrate-velocity curves of the rChAT enzyme activity at different substrate concentrations (ranging from 10 to 320 µM) with or without inhibitors at specified concentrations. The Lineweaver-Burk plots were fitted using the linear regression analysis function of GraphPad Prism 7 software. (**D**) Dose-response curves of human rChAT activity at specified substrate concentrations in the presence of different concentrations of **B1**. The IC_50_ value was calculated after fitting the curves using nonlinear regression function of GraphPad Prism 7. The values are shown as mean of two individual experiments performed in duplicate. rChAT = recombinant choline acetyltransferase.

**Figure 5:**
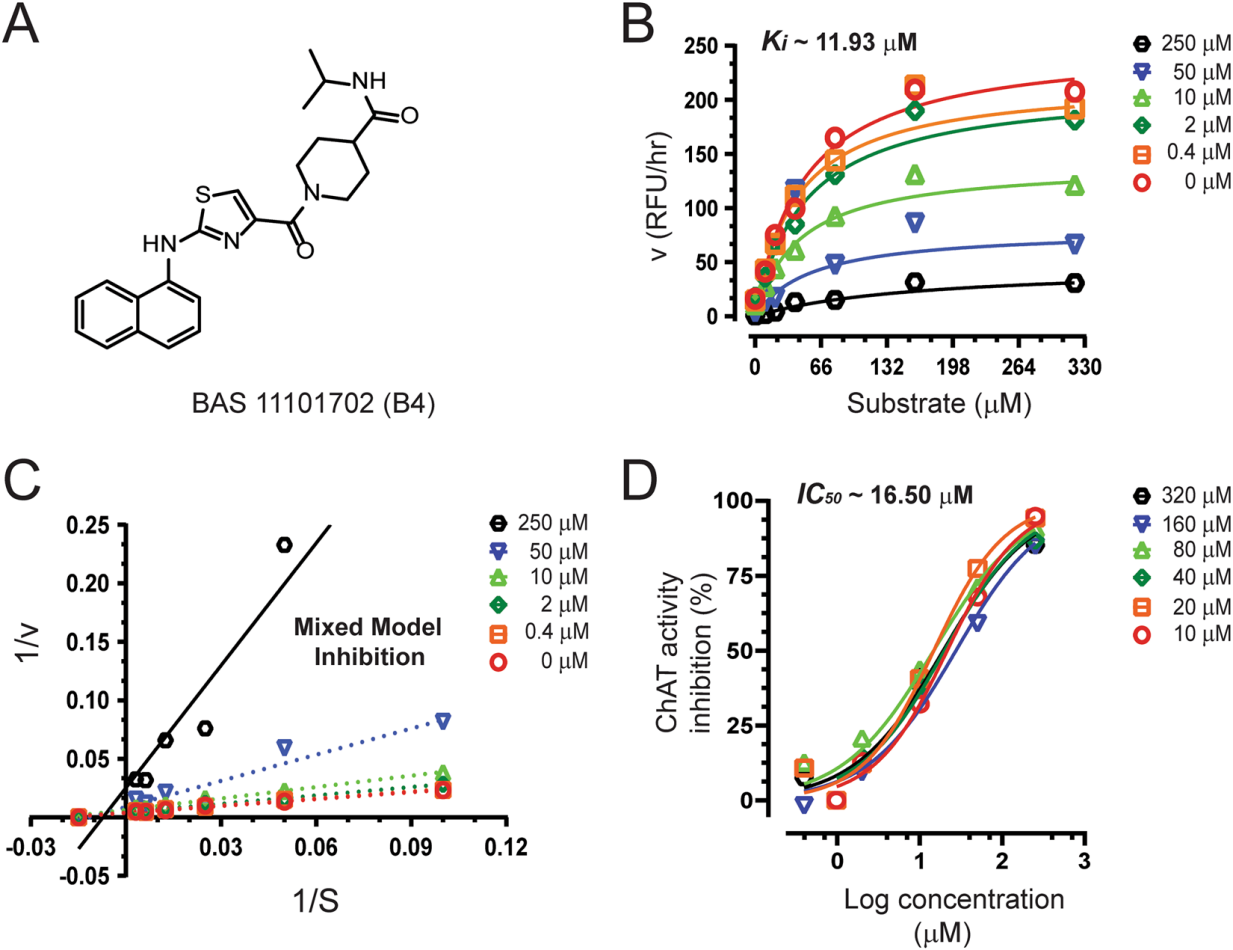
Inhibitory studies of compound **B4** against human rChAT. The specified concentrations of compound **B4** were pre-incubated with human rChAT and different substrate concentration at room temperature for 10–30 min. Afterwards; the activity was measured using our in-house non-radioactive fluorescence assay as described in the methods section. The effect of these compounds on rChAT was compared to the activity of a control sample that was pre-incubated with the buffer alone. (**A**) Structure of Asinex compound **B4**. (**B**) Showing the Substrate-velocity inhibition curves of human rChAT activity at different concentrations of **B4**. (**C**) The Lineweaver-Burk plot showing the mode of inhibition of compound **B4**. The plot was obtained from the substrate-velocity curves of the rChAT enzyme activity at different substrate concentrations (ranging from 10 to 320 µM) with or without inhibitors at specified concentrations. The Lineweaver-Burk plots were fitted using the linear regression analysis function of GraphPad Prism 7 software. (**D**) Dose-response curves of human rChAT activity at specified substrate concentrations in the presence of different concentrations of **B4**. The IC_50_ value was calculated after fitting the curves using nonlinear regression function of GraphPad Prism 7. The values are shown as mean of two individual experiments performed in duplicate. rChAT = recombinant choline acetyltransferase.

**Figure 6:**
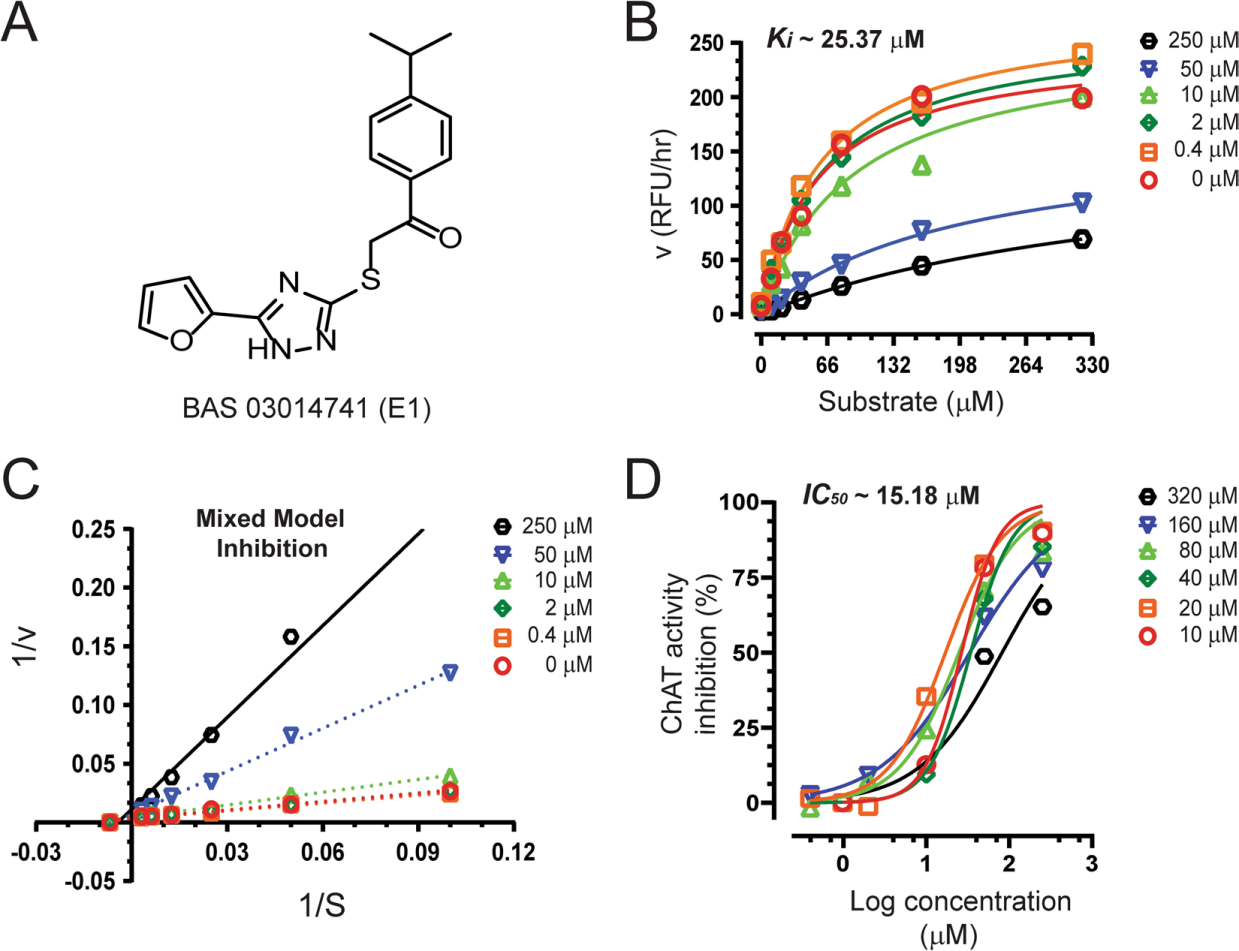
Inhibitory studies of compound **E1** against human rChAT. The specified concentrations of compound **E1** were pre-incubated with human rChAT and different substrate concentration at room temperature for 10–30 min. Afterwards; the activity was measured using our in-house non-radioactive fluorescence assay as described in the methods section. The effect of these compounds on rChAT was compared to the activity of a control sample that was pre-incubated with the buffer alone. (**A**) Structure of Asinex compound **E1**. (**B**) Showing the Substrate-velocity inhibition curves of human rChAT activity at different concentrations of **E1**. (**C**) The Lineweaver-Burk plot showing the mode of inhibition of compound **E1**. The plot was obtained from the substrate-velocity curves of the rChAT enzyme activity at different substrate concentrations (ranging from 10 to 320 µM) with or without inhibitors at specified concentrations. The Lineweaver-Burk plots were fitted using the linear regression analysis function of GraphPad Prism 7 software. (**D**) Dose-response curves of human rChAT activity at specified substrate concentrations in the presence of different concentrations of **E1**. The IC_50_ value was calculated after fitting the curves using nonlinear regression function of GraphPad Prism 7. The values are shown as mean of two individual experiments performed in duplicate. rChAT = recombinant choline acetyltransferase.

In the initial assay, compound **B1**, showed 84% inhibition of human rChAT activity as compared to only 9% and 15% inhibition for AChE and BuChE activity, respectively, indicating a high selectivity for the human rChAT enzyme (Fig. 3A; Table 2). Next, Ki and IC_50_ values were calculated from the dose-response curves at different inhibitor and substrate concentrations as shown in Fig. 4B and D. The analysis yielded a Ki value of ~9.4 µM and IC_50_ value of ~7.0 µM at substrate concentrations ranging from 20 to 80 µM. In order to determine the mode of inhibition and kinetic parameters (K_m_ and V_max_), Lineweaver-Burk (double reciprocal) plot^13^, was generated using substrate-velocity curves. In Lineweaver-Burk plots, reciprocal of rate (1/v) is plotted as a function of reciprocal of substrate concentration (1/S) for various inhibitor concentrations. The resultant plot is a straight line, with X- and Y-axis intercepts represent −1/K_m_ and 1/V_max_, respectively and the slope is K_m_/V_max_^14^. The Lineweaver-Burk plot showed an increase in K_m_ value but a decrease in V_max_ with increasing inhibitor concentrations, indicating mixed-model inhibition of human rChAT by the compound **B1** (Fig. 4C; Table 3). In addition, K_m_ and V_max_ values for each inhibitor concentration (Table 3) are also determined from the substrate-velocity curves (Figs 4B, 5B and 6B). The mixed-model inhibition mode of action of compound **B1** was confirmed by nonlinear regression fit of the data with GraphPad Prism 7^15^.

**Table 3:** The K_m_ and V_max_ values for B1, B4 and E1 hit compounds at different concentrations.

Concentration (µM)	V_max_ (µM/min)	K_m_ (µM)	R squared
**B1**			
0.0	256.6 ± 19.2	47.0 ± 10.7	0.97
0.4	247.3 ± 17.8	49.3 ± 10.7	0.97
2.0	189.6 ± 17.3	62.4 ± 16.0	0.97
10.0	154.3 ± 13.4	64.4 ± 15.5	0.97
50.0	73.5 ± 9.7	67.1 ± 24.3	0.93
250.0	54.9 ± 6.8	106.4 ± 31.3	0.97
**B4**			
0.0	254.2 ± 18.5	49.6 ± 10.8	0.97
0.4	239.0 ± 21.4	46.0 ± 12.6	0.96
2.0	226 ± 20.9	54.2 ± 14.7	0.96
10.0	150.9 ± 13.5	48.3 ± 13.1	0.96
50.0	92.1 ± 19.4	60.6 ± 40.0	0.90
250.0	45.5 ± 8.5	118.5 ± 50.5	0.94
**E1**			
0.0	249.7 ± 18.3	56.1 ± 11.9	0.97
0.4	276.1 ± 12.1	57.1 ± 11.9	0.99
2.0	265.1 ± 11.7	62.8 ± 7.7	0.99
10.0	243.8 ± 22.0	89.7 ± 20.3	0.98
50.0	167.8 ± 10.4	201.8 ± 24.0	0.99
250.0	160.3 ± 16.8	419.0 ± 67.6	0.99

Results are obtained from kinetic analysis using GraphPad Prism 7. The values are given as the mean ± SEM of two experiments.

The compound **B4** also showed high selectivity for human rChAT with 71% activity inhibition in the initial assay (Fig. 3A; Table 2). In contrast, it only showed 22% activity inhibition for BuChE and almost no inhibition of AChE activity (Fig. 3B and C; Table 2). Analogous to compound **B1**, the Ki and IC_50_ values were calculated from the dose-response curves at different inhibitor and substrate concentrations as shown in Fig. 5B and D. The Ki value of ~11.9 µM and IC_50_ value of ~16.5 µM at substrate concentrations ranging from 20 to 80 µM were obtained for **B4** from the analysis. Further, the compound **B4** affected both K_m_ and V_max_ values with increasing concentration (Table 3) and exhibited mixed-model type inhibition similar to **B1** in the Lineweaver-Burk plot (Fig. 5C) and also in nonlinear regression fit analysis.

The compound **E1**, similar to **B1** and **B4** showed high selectivity for human rChAT with 55% activity inhibition in comparison to merely 12% and 17% inhibition of AChE and BuChE activity, respectively, at the initial tested concentration (Fig. 3B and C; Table 2). Thus in the initial screening assay, the percentage inhibition of rChAT by **E1** was lower as compared to **B1** and **B4** (Fig. 3A). In addition, calculation of Ki and IC_50_ values from the dose-response curves at different inhibitor and substrate concentrations (Fig. 6B and D) resulted in ~15.2 µM and ~25.4 µM, respectively, further indicated weaker inhibition by **E1** as compared **B1** and **B4**. Surprisingly, the Lineweaver-Burk plots for **E1** (Fig. 6C) showed an exact similar pattern like **B1** and **B4**; increased K_m_ and decreased V_max_ value with increasing inhibitor concentrations (Table 3), indicating mixed-model inhibition of human rChAT, which was further confirmed by nonlinear regression fit analysis.

Overall, all three hits (**B1**, **B4**, and **E1**) showed similar behavior in the substrate-velocity curves (Figs 4B, 5B and 6B) and a dose-dependent decrease in the enzymatic rate of the human ChAT-substrate catalytic reaction (Figs 4D, 5D and 6D; Table 3). More interestingly, in spite of their different basic structural scaffold and inhibition efficiency, all three hit compounds showed a similar inhibition mechanism for human rChAT, indicating that they can bind to either enzyme or enzyme-substrate (ES) complex upon interaction, thereby interfering with the enzymatic reaction. Thus in the context of being a potential *in vivo* tracer this property may be favorable as it suggests the ligand may have access to overall available ChAT bindings sites in the brain.

### *In vitro* screening of the 35 hit compounds for selectivity over anti-targets, AChE and BuChE

**Figure 7:**
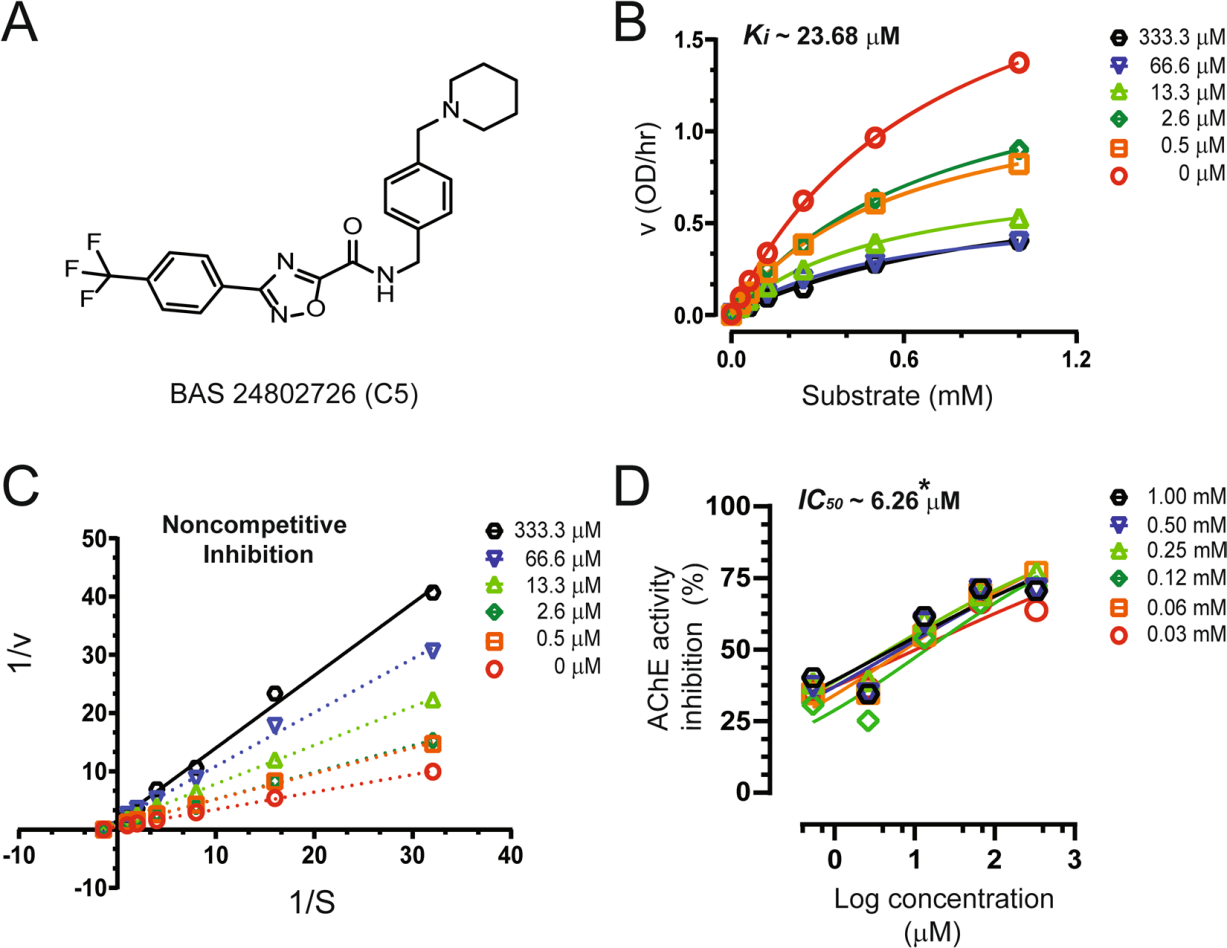
Inhibitory studies of compound **C5** against AChE. The specified concentrations of compounds **C5** were pre-incubated with AChE and different substrate concentration at room temperature for 10–30 min. Afterwards; the activity was measured using modified version of Ellman’s colorimetric assay as described in the methods section. The effect of the compound on AChE was compared to the activity of a control sample that was pre-incubated with the buffer alone. (**A**) Structure of Asinex compound **C5**. (**B**) Showing the Substrate-velocity inhibition curves of AChE activity at different concentrations of **C5**. (**C**) The Lineweaver-Burk plot showing the mode of inhibition of compound **C5**. The plot was obtained from the substrate-velocity curves of the rChAT enzyme activity at different substrate concentrations (ranging from 0.03 to 1 mM) with or without **C5** at specified concentrations. The Lineweaver-Burk plots were fitted using the linear regression analysis function of GraphPad Prism 7 software. (**D**) Dose-response curves of AChE activity at specified substrate concentrations in the presence of different concentrations of **C5**. The IC_50_ value was calculated after fitting the curves using nonlinear regression function of GraphPad Prism 7. The values are shown as mean of two individual experiments performed in duplicate. *IC_50_ value calculated at the substrate (ATC) concentration of 0.5 mM. rAChE = recombinant acetylcholinesterase.

**Figure 8:**
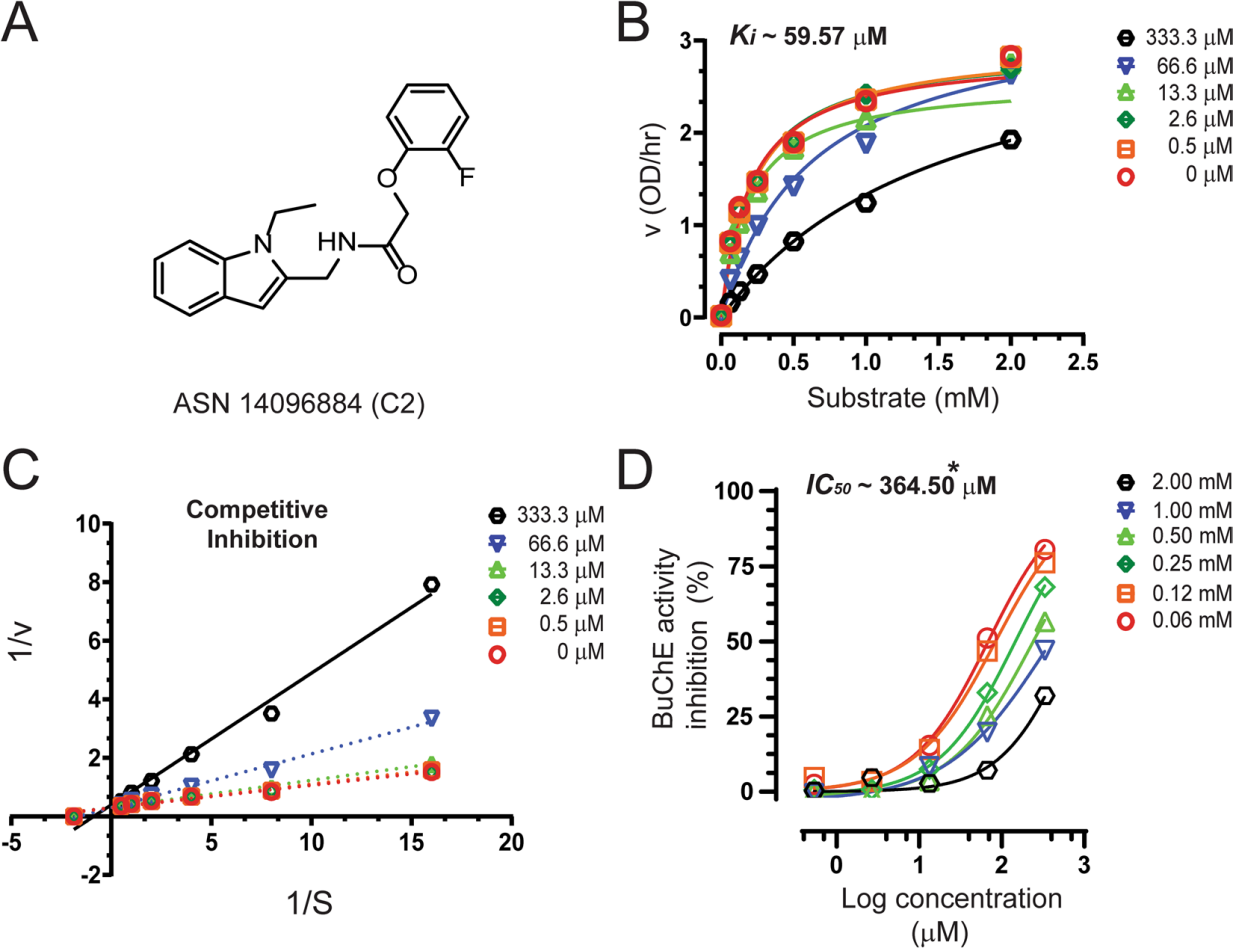
Inhibitory studies of compound **C2** against BuChE. The specified concentrations of compounds **C2** were pre-incubated with BuChE and different substrate concentration at room temperature for 10–30 mins. Afterwards, the activity was measured using modified version of Ellman’s colorimetric assay as described in the methods section. The effect of the compound on BuChE was compared to the activity of a control sample that was pre-incubated with the buffer alone. (**A**) Structure of Asinex compound **C2**. (**B**) Showing the Substrate-velocity inhibition curves of BuChE activity at different concentrations of **C2**. (**C**) The Lineweaver-Burk plot showing the mode of inhibition of compound **C2**. The plot was obtained from the substrate-velocity curves of the rChAT enzyme activity at different substrate concentrations (ranging from 0.06 to 2 mM) with or without **C2** at specified concentrations. The Lineweaver-Burk plots were fitted using the linear regression analysis function of GraphPad Prism 7 software. (**D**) Dose-response curves of BuChE activity at specified substrate concentrations in the presence of different concentrations of **C2**. The IC_50_ value was calculated after fitting the curves using nonlinear regression function of GraphPad Prism 7. The values are shown as mean of two individual experiments performed in duplicate. *IC_50_ value calculated at the substrate butyrylthiocholine (BTC) concentration of 1 mM. rChAT =  recombinant choline acetyltransferase; rAChE = recombinant acetylcholinesterase; BuChE = butyrylcholinesterase BuChE = butyrylcholinesterase.

As our main objective was to discover highly specific novel human rChAT ligands using our structure-based virtual screening method, we further screened the top 35 hit compounds for AChE and BuChE inhibitory activities in order to see whether any of the identified human rChAT hits can also inhibit either both or all of the three enzymes. We used a modified version of Ellman’s colorimetric assay^16,17^, at a single concentration of 200 µM for the inhibition assay. This initial inhibition assay identified five hit compounds for AChE (**B7**, **C1**, **C5**, **D3** and **D4**) and four for BuChE (**A2**, **C2**, **C4**, and **C5**), with more than 50% inhibition of enzyme activity at the tested concentration (Fig. 3B and C; Table 2). Like ChAT inhibitors, Ki and IC_50_ values of all nine hit compounds were calculated from the dose-response curves at different inhibitor and substrate concentrations (Table 2). The dose-response curves at different inhibitor and substrate concentrations are shown in Figs 7 and 8 for only two top hits; **C5** (for AChE) and **C2** (for BuChE).

The Ki and IC_50_ values for all these hits were in the higher micro-molar range, except for compound **C5** (for AChE, Table 2), indicating weak inhibition of both AChE and BuChE enzymes by these compounds. Moreover, out of these nine hits, only compound **C5** showed non-selective inhibition of both AChE (~82%) and BuChE (~52%) with almost no inhibition of human rChAT in the initial screening assay (Fig. 3; Table 2). The dose-response curves analysis yielded a Ki and IC_50_ value of ~23.7 µM and ~6.3 µM for AChE, respectively (Fig. 7B and D; Table 3). The IC_50_ value of **C5** for BuChE could not be calculated due to lack of complete inhibition even at the highest tested concentration of 300 µM. This clearly indicates that **C5** is a highly selective and potent inhibitor of AChE as compared to BuChE. Additionally, we also determined the **C5** mode of inhibition using Lineweaver-Burk plot, which showed a decrease in V_max_ value while K_m_ value remained almost unaffected with increasing inhibitor concentrations, indicating the non-competitive mode of inhibition of AChE by **C5** (Fig. 7C). The nonlinear regression fit of the data also suggested non-competitive inhibition (with >99% probability), further complementing the double reciprocal plot data.

On the contrary, compound **C2** showed selective inhibition of BuChE activity (78%) with no inhibitory effect on both AChE and human rChAT activity in the initial assay (Fig. 3; Table 2). The calculation of Ki and IC_50_ values from the dose-response curves at different inhibitor and substrate concentrations (Fig. 8B and D) resulted in ~59.6 µM and ~364.5 µM, respectively, suggested **C2** as a weak inhibitor as compared to other inhibitors of human rChAT and AChE identified in our studies. Further, Lineweaver-Burk plot (Fig. 8C) showed increased K_m_ value with little change in the V_max_ value with increasing inhibitor concentrations signifying the possibility of a competitive mode of inhibition, which was further confirmed by nonlinear regression analysis of the data.

Importantly, hit compounds identified as AChE and BuChE inhibitors were completely different and weaker in potency as compared to identified ChAT inhibitors and also showed a very diverse mode of inhibition. Overall, the data suggests that our virtual screening method is highly effective in discovering novel ChAT ligands with high specificity and potency.

### Cell viability assay

**Figure 9:**
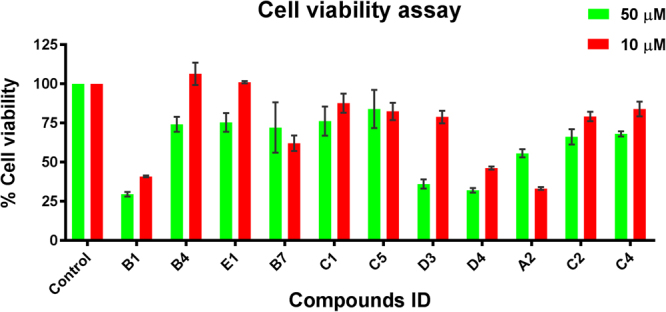
MTT based *in vitro* cell viability assay of identified active hits. Data are expressed as % mean values ± SD of 3 readings; Viability of the control cells are considered as 100%. Treatments were performed for overnight at the indicated concentrations.

Finally, to assess the prospective of the identified compounds for further development into ChAT ligands, we assessed the compounds active against ChAT, AChE and BuChE, for their cellular toxicity on human embryonic kidney (HEK 293) cells using the cell viability 3-(4,5-dimethylthiazol-2-yl)-2,5-diphenyltetrazolium bromide (MTT) assay^18^. All compounds were evaluated at 10 and 50 µM concentrations. The cell viability data is given in Table 4 (also represented as a bar chart in Fig. 9). Compounds **B1**, **B7** and **C1** reduced cell viability to less than fifty percent at 10 µM and 50 µM concentrations. Among the compounds active against ChAT, compounds **B4** and **E1** tends to be feasible for further development as they have shown lower cellular toxicity (Table 4).

**Table 4:** Cell viability assay of the active compounds.

Compound name	% of cell proliferation^a,b^	
	At 10 µM concentration	At 50 µM concentration
B1	40.9 ± 0.5	29.5 ± 1.2
B4	106.4 ± 5.8	74.2 ± 3.9
E1	101.9 ± 0.6	75.4 ± 4.9
A2	62.1 ± 4.0	72.2 ± 13.1
C2	87.7 ± 5.0	76.2 ± 7.6
C4	82.4 ± 4.5	84.0 ± 10.0
C5	78.9 ± 3.3	36.1 ± 2.4
B7	46.3 ± 0.8	32.0 ± 1.2
C1	33.2 ± 0.7	55.7 ± 2.1
D3	79.2 ± 2.5	66.2 ± 4.0
D4	84.0 ± 3.8	68.1 ± 1.4

^a^Data are expressed as % mean values ± SD of 3 readings; control cells are considered as 100%. ^b^Treatments were performed for overnight at the indicated concentrations.

### Structural analysis of active hits

#### Binding mode analysis of ChAT hits

**Figure 10:**
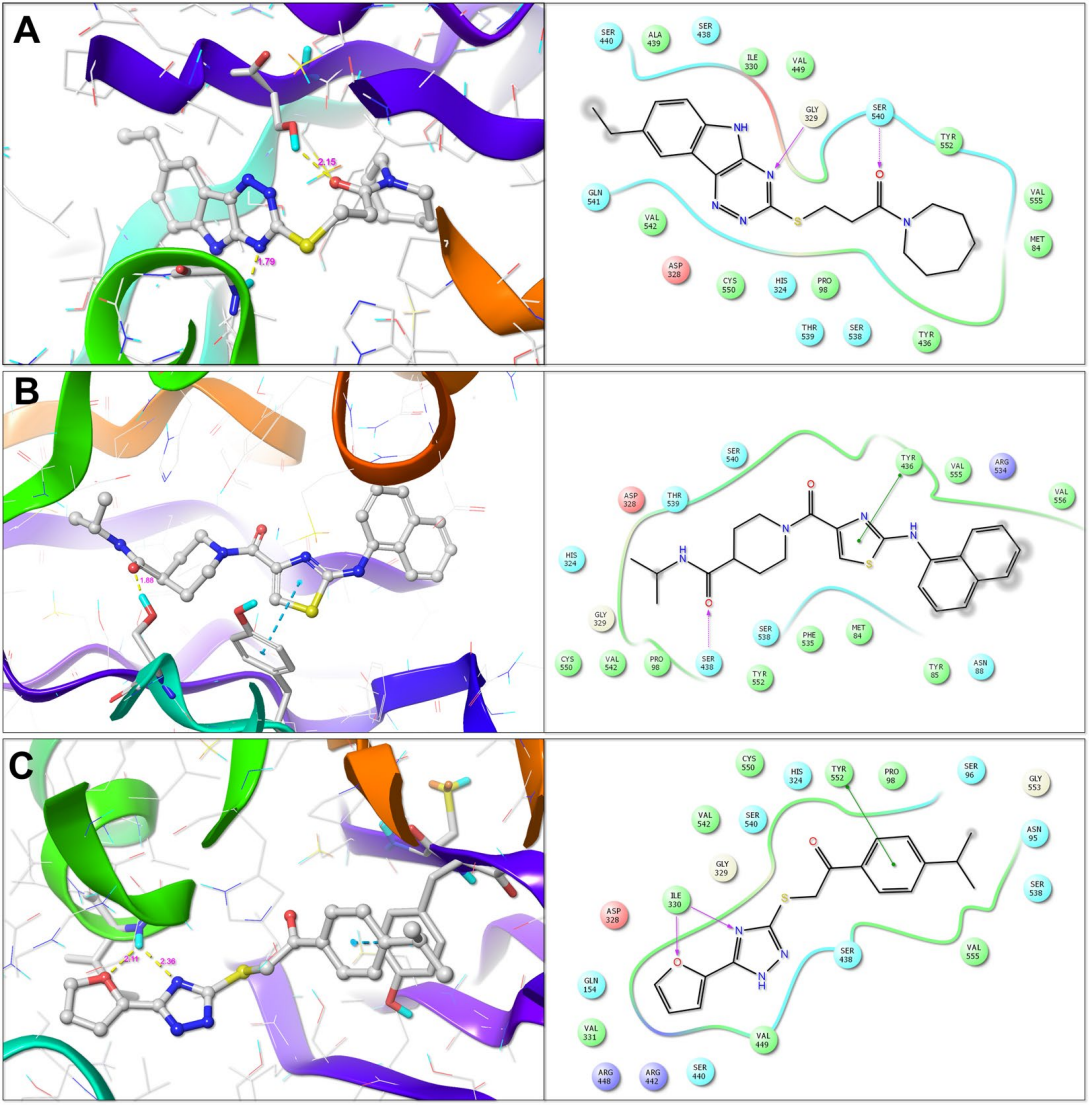
Molecular interactions of the identified hits B1 (**A**), B4 (**B**) and E1 (**C**) with active site amino acid residues of ChAT, determined using Surflex-Dock based docking protocol. The active site amino acid residues are rendered as stick and the protein backbone as ribbon. The compounds are rendered as ball and stick and the hydrogen bonds between the compound and the active site amino acid residues are shown as yellow dotted line.

The active compounds against ChAT were analyzed for their binding mode into the active site of ChAT. The docked poses of the compounds were extracted from the final step of virtual screening. The docking was performed using the ChAT crystal structure (PDB ID: 2FY3). The binding site of the enzyme was defined using co-crystallized choline coordinates. The docking scores (Total Score), which represent –logK_d_, for ChAT, AChE and BuChE is given in Table 1. The compounds **B1**, **B4**, and **E1** were identified as most active compounds against ChAT with the IC_50_ values of 7.0, 16.5 and 25.4, respectively. The three-dimensional docked pose and two-dimensional ligand interaction diagram of **B1**, **B4**, and **E1** are shown in Fig. 10(A–C). As given in the Fig. 10A, the amide carbonyl of compound **B1** formed a hydrogen bond with the SER_540_ amino acid residue of ChAT with a distance of 2.15 Å. Further, the 5H-[1,2,4]triazino[5,6-b]indole nucleus forms a hydrogen bond with the GLY_329_ amino acid residue of ChAT active site. Interestingly, the HIS_324_, which is the responsible for the catalytic transfer of acetyl group from acetyl-coenzyme A to choline for synthesis of acetylcholine is not accessible after binding of the compound **B1** (Fig. 10A), which might explain its mechanism of action.

The compound **B4** has shown a different binding mode as compared to that of **B1**. As depicted in Fig. 10B, the terminal amide carbonyl forms a hydrogen bond with SER_438_ at a distance of 1.88 Å, while the thiazole nucleus forms π -π interactions with the TYR_436_ amino acid residue of the active site of ChAT.

The compound **E1** exhibited a binding mode similar to the compound **B1**, making the HIS_324_ inaccessible for the catalysis. The triazine nitrogen and the furan oxygen form hydrogen bonds with ILE_330_ at a distance of 2.36 Å and 2.11 Å, respectively, while the benzene nucleus forms π -π interaction with TYR_552_ amino acid residue (Fig. 10C).

### Binding mode analysis of AChE and BuChE hits

**Figure 11:**
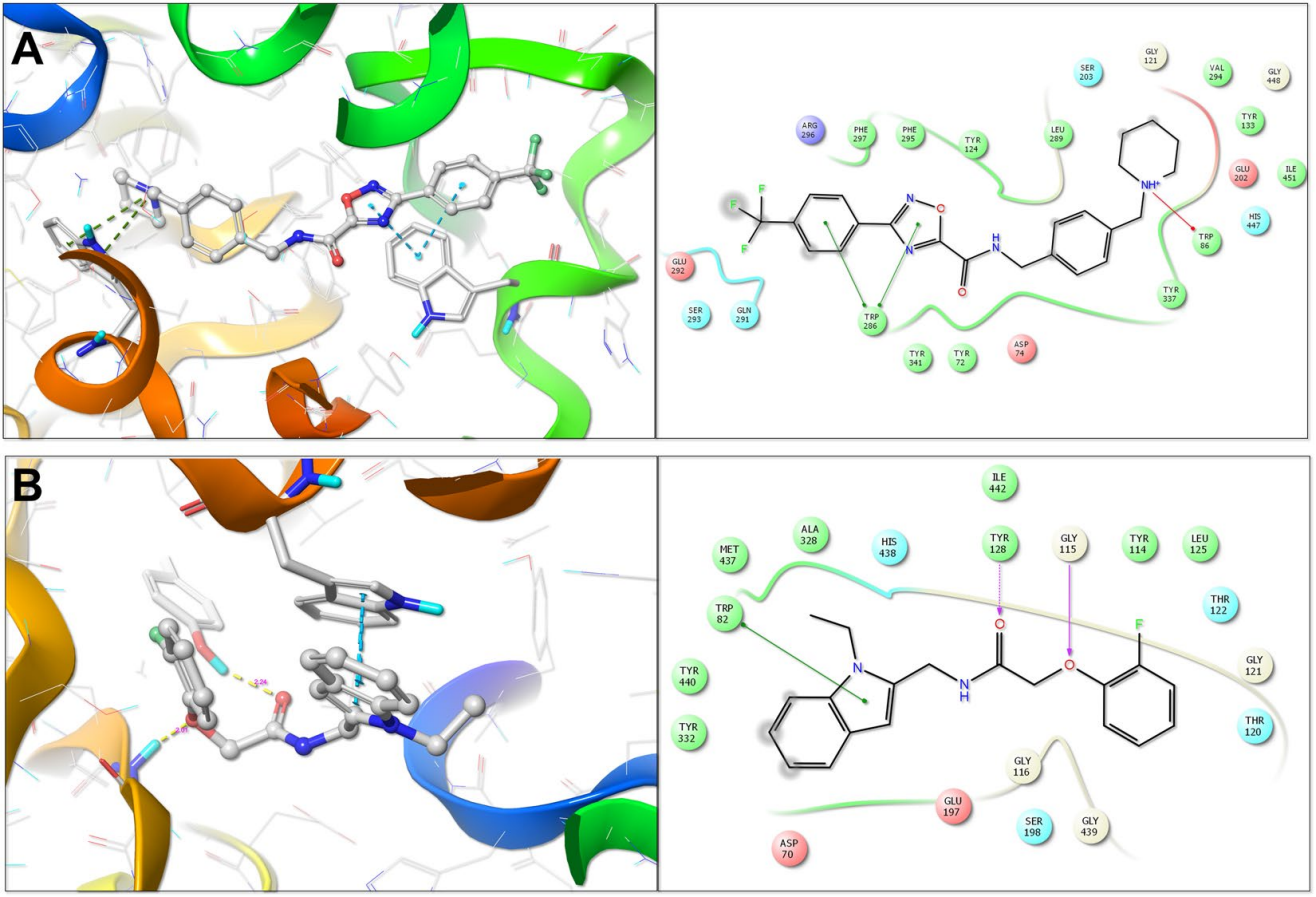
Molecular interactions of the identified hits C5 (**A**) and C2 (**B**) with active site amino acid residues of AChE and BuChE repectively, determined using Surflex-Dock based docking protocol. The active site amino acid residues are rendered as stick and the protein backbone as ribbon. The compounds are rendered as ball and stick and the hydrogen bonds between the compound and the active site amino acid residues are shown as yellow dotted line.

Of all the 35 compounds, which were evaluated *in vitro* against the anti-targets, AChE and BuChE, the compound **C5** and **C2** showed the most favorable enzyme inhibition kinetics for AChE and BuChE, respectively. We hence evaluated further their binding modes by docking them into the active site of AChE and BuCHE. The three-dimensional binding pose and the two-dimensional ligand interaction diagram for both of the compounds are given in Fig. 11A and B.

The compound **C5** bound well to the binding pocket of AChE. The oxadiazole nucleus and its adjacent benzene ring formed π -π interactions with the TRP_286_ and the piperidine nitrogen formed π -π interactions with the indole nucleus of TRP_86_ amino acid residue (Fig. 11A). Docking of compound **C2** into the active site of BuChE indicated that the indole nucleus of the compound formed π -π interactions with the indole nucleus of TRP_82_ the amide carbonyl formed a hydrogen bond with TYR_128_ at a distance of 2.24 Å, while, the oxygen atom adjacent to the benzene ring formed a hydrogen bond with the GLY_115_ at a distance of 2.01 Å.

## Discussion

Even though ChAT was discovered about a century ago^19^, surprisingly only few ChAT ligands (mostly inhibitors) are known today and they all lack the desired stability as well as brain permeability and thus have very limited applicability^9^. Here we report for the first time three new and structurally diverse ligands of ChAT. Detailed *in vitro* pharmacodynamic analyses indicated that these three ligands have considerable selectivity and potency as ChAT inhibitors.

In this study, we employed a structure based hierarchical virtual screening approach for identifying the potential novel and specific ChAT ligands with optimal BBB permeability from Asinex Gold/Platinum small molecule database. Based on our initial analysis, the top 35 compounds were selected, purchased and tested *in vitro* for their inhibitory potential against ChAT using our newly developed high-throughput non-radiometric fluorescence assay. The three identified hit compounds for ChAT had Ki and IC_50_ values in the sub-micromolar range (~7 to 25 µM).

Alzheimer’s disease is the dominating form of dementia, with a prevalent of about 50–70% of the dementia cases. Up-to-date, only four FDA-approved drugs are available on the market, which however provides symptomatic relief. Three out of these drugs are targeted at the cholinergic system which is primarily and selectively affected in AD, DLB and Down’s syndrome disorders^6,20,21^. Furthermore, most of the new drugs for AD are failing in the clinical trials phases, suggesting that early diagnosis and prevention may be pivotal for successful therapeutic intervention. Keeping in mind this urging need for novel biomarkers for early diagnosis of AD, the present work aimed at the discovery of novel ChAT ligands with the potential of radio-labeling, which can be used as an *in vivo* PET tracer to map regional brain distribution of ChAT, and thereby study the health of cholinergic network in CNS and neurodegeneration for diagnostic purposes. Given that cholinergic system is affected early in major dementia disorders, a ChAT-PET tracer may additionally have the potential for monitoring therapeutic response since an effective treatment is expected to prevent and/or to regress the functional deficit of the cholinergic network in the brain.

Amongst the identified ChAT ligands, two hits exhibited very low toxicity in the MTT assay, indicating good therapeutic window for these compounds. Although ChAT inhibitors are not suitable as AD therapeutic, they might be useful in other disorders. Intriguingly in this context, recent studies have shown a very critical role of ChAT in promoting cancer cell proliferation in both colon and lung cancer. The studies indicate that the cancer cells by upregulating ChAT may increase the synthesis of non-neuronal ACh, which act as an autocrine agent^22–24^. Their findings further suggested that any agents/therapy, which could target and stop the non-neuronal synthesis and release of ACh can be valuable in combatting disease progression. It is also possible that high ACh production and release may allow the cancer cells to avoid the immune surveillance since ACh is shown to be an effective agent for dampening the immune responses^25^. In light of these findings, the potent ChAT inhibitors identified in our study might also be developed as potential therapeutic candidates for the prevention and treatment of different forms of cancer.

The success of our hierarchical virtual screening approach was demonstrated by the identification of three ChAT ligands with high specificity, inhibition pattern, structural diversity, low toxicity, and novelty. Moreover, all these three ChAT ligands also showed a ClogP value of less than 5 indicating a good lipophilicity and CNS penetration. Importantly, the chemical scaffold of these hits can also be used as a template for designing highly selective analogs that can bind ChAT with much higher affinity and possibly be used as *in vivo* PET tracer biomarker for monitoring the possible dysfunctionality in the central cholinergic system. Such PET tracer may also be useful for diagnosis of the certain type of cancer tumors, where ChAT is overexpressed^22–24^.

These new ChAT ligands could be further improved in term of higher selectivity and potency using the in silico strategy for lead optimization of ChAT ligands that is reported in extensive details in our previous paper^26^. In addition, our newly developed ChAT assay can also be easily adapted as a high throughput *in vitro* screening method (based on 384-wells microtiter plates), with proved aptness for identification of novel and effective ChAT ligands.

Moreover, the screening of our top 35 hits for selectivity against anti-targets, AChE and BuChE, identified in total nine hits; five for AChE (**B7**, **C1**, **C5**, **D3**, and **D4**) and four (**A2**, **C2**, **C4**, and **C5**) for BuChE. Interestingly, only one hit, **C5** (out of total nine top hits) showed non-selective inhibition of both AChE and BuChE in the initial assay. On further kinetic analysis, **C5** showed a very high selectivity and an IC_50_ value of ~6.26 µM for AChE. More interestingly, all these hits, except **B7**, exhibited a high therapeutic index in the MTT toxicity assay.

Even though AChE is the main enzyme target for the current AD drug therapies, recent findings have also implicated the importance of BuChE in the later stages of disease advancement after the progressive loss of AChE in AD patients^27–29^. Thereby opening the possibility of further exploring and optimizing the highly selective AChE and BuChE inhibitors discovered in our analysis as experimental drugs for the treatment of AD.

In summary, we report for the first time, three new and potent ChAT ligands identified using our novel hierarchical virtual screening strategy, with an aim of further optimizing and developing these novel ligands into *in vivo* PET tracer biomarkers for the early diagnosis and prevention of AD and other related neurogenerative disorders.

## Methods

### Computational details

The computational studies were performed using SYBYL-X 2.1.1 (SYBYL-X 2.1.1, Tripos International, 1699 South Hanley Rd., St. Louis, Missouri, 63144, USA) molecular modeling suite installed on Linux-based Dell Precision T7610 workstation [Intel® Xeon® E5-2643 CPU @ 3.3 GHz; 16GB RAM, 2TB hard disk].

### Protein structure preparation

The 3D X-ray crystallographic structure of hChAT in complex with choline (PDB ID: 2FY3, UniProt ID: P28329) with a resolution of 2.27 Å, R-value of 0.211 and R-free value of 0.233 was downloaded from Protein Data Bank (PDB) and used for the study. It consists of residues from 120 to 733 out of a total 748 amino acid residues and has two binding pockets, one for CoA and other for choline molecule and the catalytic residue HIS_324_ lies in the middle of these two binding pockets^6^. The enzyme structure was prepared for docking prior to defining docking site by addition of any missing hydrogen, assigning a bond order, repairing side chain, treating termini, fixing protonation state and side chain amides, removal of water molecules and other ligands as implemented in prepare structure wizard of SYBYL-X suite. The co-crystallized choline substructure was extracted and finally, the energy of protein structure was minimized utilizing Powell method^30^ with Tripos force field. Similarly, the structure of AChE (PDB ID: 4EY7^31^) and BuChE (PDB ID: 4BDS^32^) were prepared and used for docking to identify selective compounds against ChAT.

### Asinex database preparation and virtual screening

Asinex Gold/Platinum collection library consisting of 296537 compounds (structure data file; SDF) was downloaded from www.asinex.com and used for the study. The library was prepared by addition of explicit hydrogens, neutralizing charged structures and generation of most probable ionization and tautomer state at pH 7.0 ± 2.0 using ligand preparation module of the SYBYL x 2.1.1 molecular modeling suite. To reduce the computational cost and increase the chance of successful identification of potential pre-clinical drug candidates, the library was screened against modified Lipinski parameters for CNS drug with potential to penetrate BBB^33^. These includes molecular weight between 150–450 dalton; cLogP <5; cLogD <4; Total polar surface area between 35–90; Hydrogen bond donors <3; Hydrogen bond acceptors <7; and Rotatable bonds <8. Further, the compounds were filtered for undesirable groups potential PAINS which make them promiscuous false positives by reacting with the screening assay method^12^. The prepared ligands were further subjected to Concord to obtain accurate and efficient, 3D global minima conformation supposed to be close to the bioactive conformation of the dataset molecules. The final dataset of 99823 compounds was obtained after preparation and was further used in the virtual screening process. The overall workflow is schematically depicted in Fig. 1.

The virtual screening process was performed in two steps using Surflex-Dock module interfaced in SYBYL-X 2.1.1 which is a fully automatic flexible molecular docking algorithm with a combination of empirical scoring function and a surface-based molecular similarity-based search engine^34^. The ‘protomol’ docking site was defined using the co-crystallized choline ligand in the active site of the enzyme. The compounds were docked into the defined active site using Surflex-Dock Screen (SFXC) for initial docking. The compounds were ranked according to their Total score and the compounds with a Total score >7 were passed to the second phase of more flexible and exhaustive docking using Surflex-Dock GeomX (SFXC). The output ligands from this step were further docked to the active site of AChE and BuChE as an additional check to identify only selective ChAT ligands. Finally, 35 compounds were selected based on their low total score against AChE and BuChE and high total score against ChAT, and importatnt interactions with the active site amino acid residues of ChAT. The selected compounds were purchased commercially for *in vitro* testing using our in-house developed non-radioactive enzymatic ChAT assay method.

### Purification of recombinant ChAT

DYT media (16 g/l Tryptone, 10 g/l yeast extract, 5 g/l NaCl, 100 µg/ml ampicillin, 34 µg/ml chloramphenicol) was inoculated with a preculture of E.Coli BL21 Rosetta2 transformed with pProEXHTa-ChAT (a gift from Dr. Brian Shilton, Department of Biochemistry, University of Western Ontario, London, Canada). The bacteria were grown in shaking incubator at 37 °C with 200 rpm until the optical density at 600 nm reached 0.5. After which, 0.5 mM IPTG was added and His_6_-ChAT was expressed for circa 16 h at 18 °C. The bacteria were harvested and stored at −80 °C.

His_6_-ChAT was purified with “Ni-NTA fast start Kit” (Qiagen) following the manufacturer’s instructions. The elution buffer was exchanged to storage buffer (10 mM Tris pH 7.4, 500 mM NaCl, 10% (v/v) glycerol) using 30 kDa molecular weight cutoff Amicon Ultra concentrators (Merck Millipore). The purified protein was aliquoted, and stored at −80 °C. The purity and molecular weight of the protein was determined using SDS-PAGE electrophoresis. The total protein concentration was measured with DC Protein Assay (BioRad).

### *In vitro* AChE and BuChE activity inhibition assay

A modified version of Ellman’s colorimetric assay^16,17^ was adapted to a high throughput assay for the enzymatic activity of BuChE and AChE. The reagent, butyrylthiocholine iodide (BTC), acetylthiocholine iodide (ATC), 5,5′-dithiobis (2-nitrobenzoic acid) (DTNB) were purchased from Sigma-Aldrich (St. Louis, MO, USA). The modified assay details are as previously described^34,35^. The main modification concerned with the high throughput adaptation of the assay for use in 384-well plates. Briefly, 25 μL/well of a 1:450 diluted solution of a pooled human plasma and 1:768 diluted (3.5 ng/ml final concentration) purified recombinant human AChE protein (Sigma, Cat no. C1682) was used for measurement of BuChE and AChE activity, respectively. In the initial *in vitro* screening step, the wells were pre-incubated with 25 μL/wells of different Asinex compounds for 10–30 minutes at room temperature. The concentration of the compounds working solution was 3X to give a final concentration of 200 μM in a final volume of 75 μL in each well. The stock concentration of the compounds were  prepared in 100% DMSO. The stock concentration was chosen usually at 10 mM, allowing the final DMSO concentration in the wells to be less than 4%. Finally, 25 μL of a cocktail mix prepared in Na/K phosphate buffer, containing DTNB (final concentration 0.4 mM) and BTC, (final concentration 1 mM) or ATC (final concentration 0.5 mM) was added to each well and the changes in absorbance was monitored at 412 nm wavelength for 15–20 minutes with one-minute interval, using a microplate spectrophotometer reader (Infinite M1000, Tecan). The rate of the enzyme activity was determined from the linear part of the kinetic reaction curves as ∆OD/time.

### *In vitro* ChAT activity inhibition fluorometric assay

ChAT activity was measured using our newly developed fluorometric assay, using human recombinant ChAT (rChAT) protein. The reagents, choline chloride, acetyl coenzyme-A (ACoA, A2181) and 7-Diethylamino-3-(4-maleimidophenyl)-4-methylcoumarin (CPM) were purchased from Sigma-Aldrich (St. Louis, MO, USA).

The ChAT assay could be run in either 96-well or 384-well plates. For 96-well plates, 50 μL/well of 0.212 µg/ml (final concentration) of the recombinant ChAT was incubated with 100 μM different Asinex compounds (50 μL/well) for 10–30 minutes at room temperature in dilution buffer (10 mM Tris-HCl, pH 7.4, 150 mM NaCl, 1.0 mM EDTA, 0.05% (v/v) Triton X-100). Then, 50 µL of a cocktail-A [dilution buffer containing choline chloride (final concentration 150 µM), ACoA (final concentration 13.3 µM) and CPM (final concentration 15 µM)] was added to each well. Immediately after adding the cocktail-A, the changes in fluorescence was monitored kinetically at 479 nm after exciting at 390 nm at 1–2 minute intervals for 15–20 minutes using a microplate spectrophotometer reader (Infinite M1000, Tecan).

Each Asinex compounds were run in triplicates. On each 96-wells plate, several enzyme wells without inhibitor were also included during measurements as control and for estimating the inhibition level. Negative controls were wells without enzyme. The percentage inhibition for each Asinex compound was calculated based on the enzyme control value as a reference (100% activity). The compounds showing over 50% inhibition of enzyme activity were selected for further kinetic studies.

### Kinetic studies or *in vitro* estimation of Ki, IC_50,_ and mode of action of hits

For kinetic studies, a similar protocol as inhibition assay was followed; a dilution series of five different concentrations ranging from 10^−6^ to 10^−9^ M were prepared for each selected Asinex compounds. For ChAT, the A-CoA was kept constant at 10 μM (final) but the concentration of choline chloride varied between 320–10 μM. For BuChE, BTC was used as the substrate in the concentrations ranging from 0.0625 to 2 mM. For AChE, ATC was used as the substrate in the concentrations ranging from 0.0312 to 1 mM. Each compound concentration was measured in duplicates. The rate of enzyme activity (as ∆OD/hr kinetic data) was calculated and processed using the GraphPad Prism 7 analysis software^15^. The inhibitory constant (Ki) values were determined from the dose-response curve and the half-maximal inhibitory concentration (IC_50_) values were calculated by plotting the percentage enzyme activity vs. the log of the Asinex compound concentrations and fitting the data using the nonlinear regression Enzyme Kinetics-Inhibition function.

The Michaelis-Menten constant (K_m_) and maximal velocity (V_max_) values were calculated from substrate-velocity curve after fitting the data with non-linear regression Michaelis-Menten kinetic function. The K_m_ and V_max_ values were further used to plot the Lineweaver-Burk plots; the plots were fitted using linear regression function.

### Cell viability assay

The *in vitro* cellular toxicity of the active compounds was evaluated using MTT assay^18^. Briefly, Human embryonic kidney epithelial (HEK-293) cells cultured at 37 °C in a humidified environment with 5% CO_2_ in Dulbecco’s Modified Eagle’s medium (DMEM) in 96 well plate to obtain 70–80% confluency. Further, the cells were incubated overnight at 37 °C with 10 µM and 50 µM compounds in triplicate. Cells treated with 1% DMSO were used as controls. At the end of the treatment (12 h), 10 μL of 12 mM MTT dissolved in Phosphate buffer saline pH 7.4, was added to each well and incubated for an additional 2 h at 37 °C. The media was then removed from the plate and 100 µL of DMSO was added to dissolve the violet formazan crystals. Finally, the absorbance was measured at 540 nm with a reference wavelength of 630 nm on Tecan spectrophotometer with shaking before the reading. The percentage viability was calculated taking 1% DMSO control as 100 percent and presented as mean ± SD.
